# Relevance of Post-Stroke Circulating BDNF Levels as a Prognostic Biomarker of Stroke Outcome. Impact of rt-PA Treatment

**DOI:** 10.1371/journal.pone.0140668

**Published:** 2015-10-15

**Authors:** Marion Rodier, Aurore Quirié, Anne Prigent-Tessier, Yannick Béjot, Agnès Jacquin, Claude Mossiat, Christine Marie, Philippe Garnier

**Affiliations:** 1 Unité INSERM U1093 Cognition, Action et Plasticité Sensorimotrice, Dijon, F-21078, France; 2 Université de Bourgogne Franche-Comté, Dijon, F-21079, France; 3 Département Génie Biologique, IUT, Dijon, F-21078, France; 4 Department of Neurology, University Hospital, Dijon, F-21000, France; 5 Centre d’Epidémiologie des Populations, EA4184, Dijon, F-21000, France; Centre national de la recherche scientifique, University of Bordeaux, FRANCE

## Abstract

The recombinant form of tissue plasminogen activator (rt-PA) is the only curative treatment for ischemic stroke. Recently, t-PA has been linked to the metabolism of brain-derived neurotrophic factor (BDNF), a major neurotrophin involved in post-stroke neuroplasticity. Thus, the objective of our study was to investigate the impact of rt-PA treatment on post-stroke circulating BDNF levels in humans and in animals. Serum BDNF levels and t-PA/plasmin activity were measured at hospital admission and at up to 90 days in stroke patients receiving (n = 24) or not (n = 14) rt-PA perfusion. We investigated the relationships between serum BDNF with concurrent t-PA/plasmin activity, neurological outcomes and cardiovascular scores at admission. In parallel, serum BDNF levels and t-PA/plasmin activity were assessed before and after (1, 4 and 24h) the induction of ischemic stroke in rats. Our study revealed higher serum BDNF levels and better neurological outcome in rt-PA-treated than non-treated patients. However, serum BDNF levels did not predict stroke outcome when the whole cohort of stroke patients was analyzed. By contrast, serum BDNF levels when measured at admission and at day 90 correlated with cardiovascular scores, and those at day 1 correlated with serum t-PA/plasmin activity in the whole cohort of patients whereas no association could be found in the rt-PA-treated group. In rats devoid of cardiovascular risk, no difference in post-stroke serum BDNF levels was detected between rt-PA- and vehicle-treated animals and no correlation was found between serum BDNF levels and t-PA/plasmin activity. Overall, the data suggest that serum BDNF levels may not be useful as a prognostic biomarker of stroke outcome and that endothelial dysfunction could be a confounding factor when serum BDNF levels after stroke are used to reflect of brain BDNF levels.

## Introduction

Despite intensive preclinical research that has led to a better characterization of the complex pathogenesis of stroke, the only available curative pharmacological treatment for stroke patients is the recombinant form of the tissue plasminogen activator (rt-PA). According to its primary function, rt-PA converts fibrin-bound plasminogen into active plasmin, which in turn dissolves the fibrin of blood clots, thus achieving arterial recanalization in acute ischemic stroke. However, considerable experimental evidence has shown that beyond its fibrinolytic function, t-PA is implicated in many different processes [1]. In physiological conditions, t-PA is involved in neuronal migration and synaptic outgrowth during development while in the adult brain, t-PA is implicated in neurotransmission, synaptic plasticity and cognitive function [2–4]. In addition, experimental studies have revealed that t-PA could have adverse effects and acts as a pro-hemorrhagic, pro-inflammatory and pro-excitotoxic factor in pathological conditions [5]. The pleiotropic effects of t-PA, which is able to cross blood brain barrier [6] can be explained by the activation of numerous receptors expressed by cells of the neurovascular unit [7] and also by direct or indirect (plasmin) proteolytic action. Recently, particular attention has been paid to the potential relationship between t-PA (either endogenous or exogenous) and brain-derived neurotrophic factor (BDNF), a signaling molecule that is crucial in adaptive neuroplasticity [8–10]. Indeed, it has been established that t-PA through plasmin activation is involved in the cleavage of proBDNF into its mature form [11,12] and that exogenous t-PA increases mature BDNF expression in the hippocampus through N-methyl-D-aspartate (NMDA) receptor activation [13].

BDNF is present in the blood [14] and circulating BDNF is widely used as an indicator of brain BDNF levels by neurologists and psychiatrists [15,16]. Although the correlation between brain and circulating BDNF levels has not always been established in animal studies [17,18], the measurement of circulating BDNF appears to be of great interest for the diagnosis, the prognosis and treatment monitoring of various diseases of the central nervous system. Surprisingly, there is a lack of studies specifically designed to investigate the impact of t-PA treatment on circulating BDNF levels in stroke.

In this context, the objective of our study was to compare serum BDNF levels in rt-PA-treated and non-treated stroke patients and to investigate mechanisms involved in potential difference. We therefore assessed the relationships between serum BDNF levels at admission and at up to 90 days and i) concurrent serum t-PA/plasmin activity, ii) the neurological status assessed from the NIHSS score and iii) the cardiovascular score at admission. In parallel, serum BDNF levels and t-PA/plasmin activity were assessed before and after (1, 4 and 24h) the induction of ischemic stroke in rats. These results may help to evaluate the relevance of circulating BDNF as a prognostic biomarker in stroke patients.

## Material and Methods

### Clinical study

#### Setting, standard protocol approval, registrations and patients consents

This prospective longitudinal study was conducted at Dijon University Hospital, from September 2010 to April 2012. Recruited patients were of both genders and admitted to the stroke unit department for ischemic stroke and treated or not with intravenous (i.v.) rt-PA (0.9 mg/kg, Actilyse, Boehringer Ingelheim). Treatment allocation was based on current guidelines on the use of rt-PA in acute ischemic stroke. Reasons for non-administration of rt-PA were exceeded time window or other contra-indications such as pre-stroke use of anticoagulants. Informed consents was given by the patients themselves or by a close relative. Information about the study was given orally to each participant by doctors involved in the study. A document explaining the protocol was systematically provided. This document was approved by the Comité de Protection des Personnes (Committee for the protection of people participating in research studies), which is the legal ethics committee that approves human studies in France. Following the recommendations of this committee, only a verbal consent was required from patients. Participants were listed by the Research Department of the University Hospital of Dijon. The study was approved by the health authorities and relevant ethical committee: Comité de Protection des Personnes (CPP) Nord Est I Dijon, France, by 11-18-2010, under registration number 2010-A01181-38.

#### Inclusion and non-inclusion criteria

Stroke patients aged over 18 years of age with a National Institute of Health Stroke Scale (NIHSS) score between 4 and 25 at hospital admission for ischemic stroke (less than 12h after onset) and who had given their consent to participate were eligible. Non-inclusion criteria were (1) hemorrhagic stroke, (2) transient ischemic attack (TIA), (3) aphasia before and at the time of stroke, (4) prior dementia (5) impairment of daily living before stroke onset with a pre-stroke modified Rankin Scale score > 4, (6) depression and (7) pregnancy.

#### Blood samples

Each patient provided four blood samples. The first blood sample (D0) was taken in the neurovascular emergency department after consent and before the rt-PA treatment for treated patients (rt-PA-treated). The second and third samples were obtained at 8 am the next day (D1) and on the seventh (D7) day after admission. The last sample (D90) was collected during the inspection visit, about 90 days after admission.

#### Clinical assessment

The demographic data (gender and age), the lesion location and the conventional cardiovascular risk factors (hypertension, diabetes, hypercholesterolemia, smoking and alcohol abuse) were obtained. The etiology of stroke was classified using TOAST (Trial of ORG 10172 in Acute Stroke Treatment) criteria. Stroke severity was assessed at admission and at 1, 7 and 90 days after stroke onset using the NIHSS score assessed by a neurologist. The cardiovascular health status was determined using the European Society of Cardiology (ESC) scale (http://www.escardio.org/communities/EACPR/toolbox/health-professionals/Pages/SCORE-Risk-Charts.aspx). This scale is based on gender, age, total cholesterol, systolic blood pressure and smoking status. Of note, the cardiovascular score was calculated using the systolic blood pressure measured at admission. Consequently, the score could be overestimated. However, no difference in serum BDNF levels was observed between patients with a high (>160 mm Hg) or a low (<160 mm Hg) systolic blood pressure (Table 1). Of note, no difference was observed when the 185 mm Hg limit was used either (data not shown). In addition, no correlation was observed between systolic blood pressure assessed at admission and BDNF levels measured at the different time points (S1 Table).

**Table 1:** Serum BDNF levels in stroke patients with a systolic blood pressure lower or higher than 160 mmHg at the admission.

	Serum BDNF levels (pg/ml)		
	Patients with systolic blood pressure <160mmHg at admission (mean ± SEM) n = 13	Patients with systolic blood pressure >160mmHg at admission (mean ± SEM)n = 21	P value^a^
**D0**	11348.62 ± 1256.89	9823.75 ± 1036.82	0.516
**D1**	9145.06 ± 989.54	8476.75± 1155.01	0.577
**D7**	8116.9 ± 1037.04	7124.5 ± 1521.45	0.534
**D90**	7876 ± 1262.53	6177.5 ± 1260.62	0.424

Levels were measured at D0 (admission), D1, D7 and D90 after ischemic stroke. Values are expressed as means ± SEM.

^a^ Comparisons between the two groups of patients were analyzed at the different time points using the non-parametric Mann–Whitney-U test with significance set at (*) p <0.05.

#### Study issues

The primary endpoint is based on serum levels of BDNF in venous blood samples (1) at different times. Secondary endpoints were t-PA and plasmin activity in serum (2), the platelets count in the different groups of patients, (3) the NIHSS score (4) and the cardiovascular health status by the ESC score (5).

### Preclinical study

#### Animals

The experiments were carried out on adult male Wistar rats (290–310 g; Janvier Labs, Saint Berthevin, France, n = 22), conducted according to the French Department of Agriculture guidelines (license 21CAE099) and performed in order to comply with the ‘Animal Research: Reporting In Vivo Experiments’ ARRIVE guidelines. The animals were housed five per cage and kept under a 12/12h light/dark cycle with *ad libitum* access to food and water. The experimental procedures were approved by the committee for ethics in animal experimentation (agreement 6112) of Animal Housing Facility of the University of Burgundy. Every effort was made to minimize animal suffering and to reduce the number of animals used. Body temperature was monitored and maintained at 36–38°C during the surgical procedure. The choice of dose and the route of rt-PA administration were made in agreement with the literature. All of the treated animals were included in this study. In order to minimize the impact of circadian cycle, the animals were homogeneously allocated in each group with respect to the time of day.

#### Ischemic stroke model

The rats were subjected to a permanent focal ischemia induced by photothrombotic occlusion of cortical microvessels, a model routinely used in our laboratory [19–21]. Briefly, anesthesia was induced by intraperitoneal (i.p.) injection of chloral hydrate (400 mg/kg). Then, the anesthetized rats were infused for 20s with the photosensitizer dye rose bengal (20 mg/kg, i.v.) and a laser beam was focused with an optical fiber (1 mm internal diameter, emerging power 90 mW) through the skull on the right hemisphere (-0.5 mm posterior and 3.5 mm lateral relative to the bregma). The laser system was a diode-pumped solid-state laser (LCS-DLT-312, Opton Laser International, Orsay, France) working at 532 nm. The skull was irradiated for 5 min, the irradiation beginning 30s before the dye injection. Using this site of irradiation, infarction was confined to the motor cortex. Twenty-four hours following ischemia, the animals were anesthetized by i.p. injection of chloral hydrate (400 mg/kg) and euthanized.

#### Pharmacological treatment

After ischemia, the animals were perfused with vehicle (L-Arginin 3.5% in deionized water, n = 8) or rt-PA (Actilyse, Boehringer Ingelheim) 10 mg/kg i.v. (undialyzed, n = 8) as a bolus (10% of total dose) followed by a 60-min perfusion (90% of total dose) using pump infusion (Harvard Apparatus, 55–4150) to mimic the clinical protocol of rt-PA administration.

#### Blood samples

Four blood samples were taken from each rat by venous puncture of the left jugular vein. The blood samples were taken before and after (1, 4 and 24h) stroke induction. The first, second and last samples were taken under chloral anesthesia (400 mg/kg) while the third was collected under short-term halothane anesthesia in order to reduce the overall duration of the anesthesia.

### Biochemical measurements

#### BDNF measurement

Total BDNF levels were determined with a commercial sandwich ELISA kit (ChemiKine™, MERCK MILLIPORE). For this kit, rabbit polyclonal antibodies coated onto plates were generated against human BDNF and the captured BDNF were detected using biotin conjugated mouse monoclonal antibodies. BDNF antibodies do not cross with NGF, NT 4/5 or NT3. The limit of sensitivity was 6 pg/mL. The experimental procedure was performed according to the manufacturer's instructions. Serum samples were diluted (1/10, v/v) in TRIS buffer (pH 7.4). The diluted samples (50 μL) were again diluted (1/2, v/v) directly on the plate in a buffer provided by the kit. All assays were performed in duplicate. Circulating BDNF levels were expressed as pg/mL.

#### t-PA and plasmin activities assay

t-PA and plasmin activities were measured using Sensolyte® AMC t-PA Activity Assay and Sensolyte® Rh 110 Plasmin Activity Assay (Anaspec, TEBU Bio, Le Perray-en-Yvelines, France), respectively. Experiments were performed according to the manufacturer's protocol. Briefly, 50 μl of fluorimetric substrate was incubated with 50 μl of serum diluted in saline 1/50 (v/v) for t-PA activity and 1/10 (v/v) for plasmin activity. Measurements were made in duplicate at 30°C over a 60-min period using a multiplate reader (Wallac Victor^2^ 1420 Multilabel Counter). Relative fluorescent unit (RFU) per min were normalized by protein concentration.

### Statistical analysis

The results are expressed as means ± standard error of the mean (SEM). All statistical analyses were done using systat 9.0 software (SPSS Science SoftwareGmb, Erkrath, Germany). Differences between two groups were assessed at the different time points using the non-parametric Mann–Whitney-U test with the significance set at p <0.05.

To investigate the relationship between two variables, a Shapiro-Wilk normality test (significance level p <0.05) was used on populations. If the population distribution was normal, the search for a correlation between the two parameters was evaluated by using the Pearson correlation test (significance level p <0.05). If the distribution was not normal, the Spearman rank correlation test (significance level p <0.05) was used. For the analysis of population characteristics (demographic characteristics, lesion location, risk factors, etiology and death), the two groups were compared using an χ2 test to which the Yates correction was applied.

## Results

### Clinical study

#### Characteristics of ischemic stroke patients

Among the recruited patients (n = 40), 25 received rt-PA treatment within the first 4.5h after stroke onset. Except for the mean time interval between stroke onset and D0 blood samples realized just before the rt-PA treatment for treated patients, there were no significant differences between the 2 groups for any of the parameters (Demographic characteristics, lesion location, stroke etiology, cardiovascular risk factor, NIHSS) presented in Table 2. Among the recruited patients, two were excluded from the study analysis (for hysterical conversion and because of early systemic hemorrhage within the first 24h).

**Table 2:** Population characteristics in non-treated and in rt-PA treated patients.

Variables	Non-treated Patients (n = 14)	rt-PA-treated Patients (n = 24)	P value
**Demographic Characteristics**			
Sex (M/F)	8/6	5/9	
Age (years)	74.71±3.55	69.13±3.01	NS ^a^
BMI (kg/m^2^)	26.11±0.84	25.00±5	NS ^a^
**Mean time interval between stroke onset and D0 blood samples (min)**	358± 60.57	165 ± 13.25	0.007 *^a^
**Lesion Location**			
Left Hemisphere	9	16	NS ^b^
Right Hemisphere	5	9	NS ^b^
Both Hemispheres	1	0	-
**Stroke Etiology**			
Hypertension	7	14	NS ^b^
Diabetes	1	9	NS ^b^
Hypercholesterolemia	1	5	NS ^b^
Smoking	3	6	NS ^b^
Alcoholism	3	4	NS ^b^
(Heart failure / Atrial fibrillation / Myocardial infarction history)	5	7	NS ^b^
**NIHSS**			
D0 (mean ± SEM)	11.44±2.25	11.20±1.34	NS ^a^
D1 (mean ± SEM)	11.67±3.13	8.17±1.75	NS ^a^
D7 (mean ± SEM)	9.43±2.53	5.60±1.64	NS ^a^
D90 (mean ± SEM)	3.86±1.51	2.00±0.97	NS ^a^
**Death**	2	5	NS ^b^

Population characteristics in non-treated patients (n = 14) and in rt-PA-treated patients (n = 24). Values are expressed as mean ± SEM or numbers of patients (Lesion location, stroke etiology, cardiovascular risk factors, death). BMI = Body mass index, NIHSS = National Institute of Health Stroke Scale, NS = non-significant.

^a^ Differences between the two groups of patients were analyzed using the non-parametric Mann–Whitney-U test with significance set at (*) p <0.05.

^b^ Differences between the two groups of patients were assessed using a χ2 test to which the Yates correction was applied.

#### Post-stroke serum BDNF levels and platelet count

**Fig 1:**
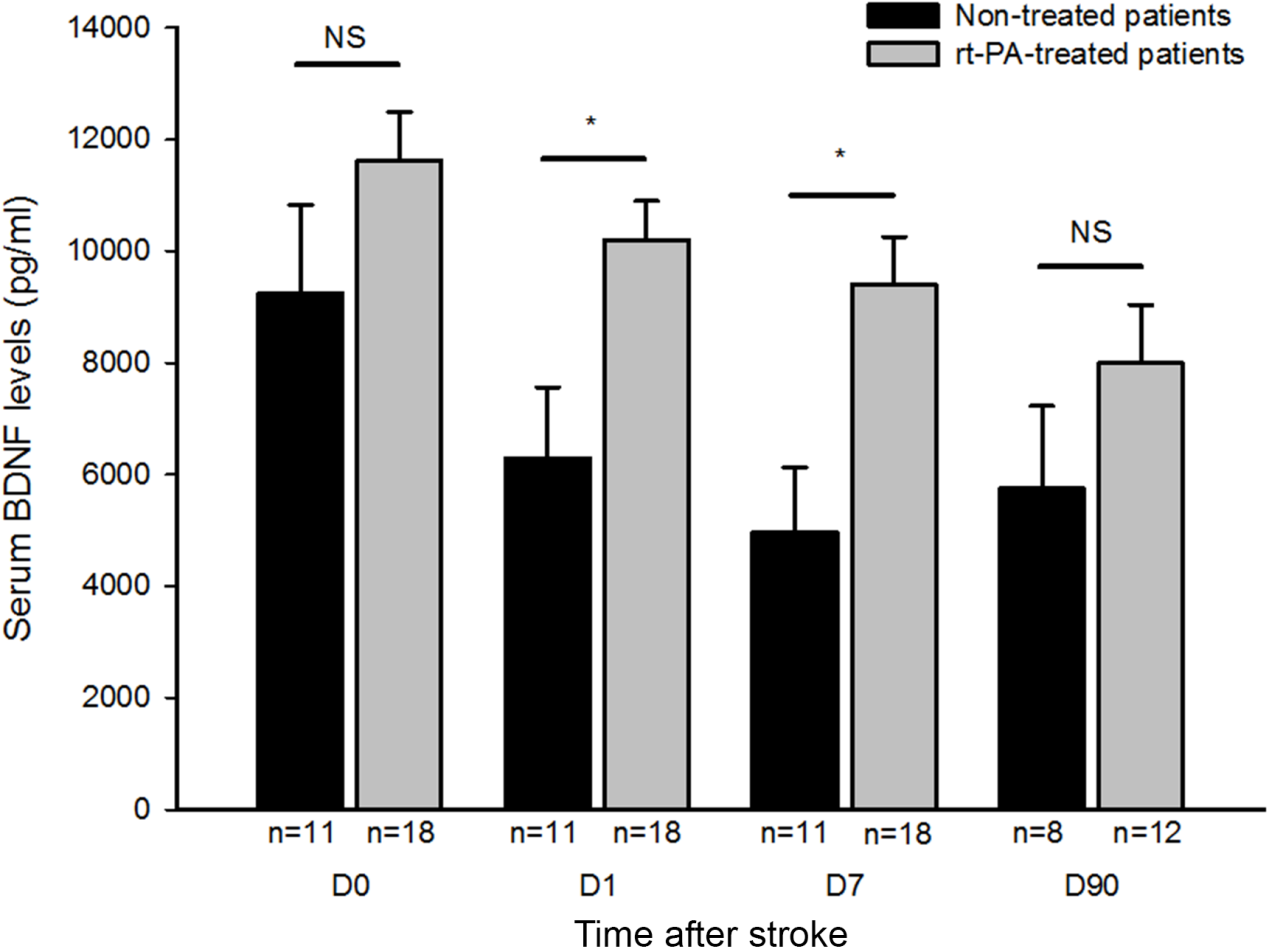
Serum BDNF levels in stroke patients assessed at D0 (admission), D1, D7 (non-treated, n = 11 and rt-PA-treated, n = 18) and D90 (non-treated, n = 8 and rt-PA-treated, n = 12) after ischemic stroke. Values are expressed as mean ± SEM. ^a^ Differences between the two groups of patients were analyzed at the different time points using the non-parametric Mann–Whitney-U test with significance set at (*) p <0.05.

In the analysis of the two group of patients (Fig 1), serum BDNF levels at days 1 and 7 were significantly higher in rt-PA-treated than in non-treated patients (p = 0.024 and p = 0.021, respectively). Please note that at 90 days the number of patients dropped due to patient death and difficulties in the patient following at this belated time point. In order to verify whether or not these differences in serum BDNF levels were due to changes in thrombocythemia, the platelet count was assessed from day 0 to day 7 (Table 3). No difference in the number of platelets was observed between rt-PA-treated and non-treated patients. In addition, to verify whether the time to treatment interval could have affected serum BDNF levels, patients treated 2h after stroke onset were dissociated from patients treated beyond 2h. These results gathered in S2 Table showed that there was no difference at admission (D0) and D1 between patients treated soon after stroke and patients treated after a longer delay.

**Table 3:** Comparison of platelet counts in stroke patients treated or not with rt-PA.

	Number of platelets (Giga/l)		
	Non-treated patients	rt-PA-treated patients	P value^a^
D0	213.27 ± 15.57	241.38 ± 24.12	0.796
D1	219.33 ± 11.76	218.18 ± 11.91	0.916
D7	232.87 ± 14.66	243.78 ± 25.18	1
D90	-	-	

Platelet counts in the blood of stroke patients (non-treated, n = 11 and rt-PA-treated, n = 21) was assessed at D0 (admission), D1 and D7 after ischemic stroke. Values are expressed as mean ± SEM.

^a^ Differences between the two groups of patients were analyzed at the different time points using the non-parametric Mann–Whitney-U test with significance set at (*) p <0.05.

#### Post-stroke t-PA/plasmin activity and correlations between serum BDNF levels and t-PA/plasmin activity

Serum t-PA and plasmin activity (Table 4) were measured from day 0 to day 90. No significant difference in t-PA and plasmin activity was observed between the two groups of patients at the different time points. Although rt-PA-treated patients exhibited higher t-PA and plasmin activity, the difference between the two groups of patients did not reach significance. As expected, in the whole cohort of patients, plasmin activity correlated significantly to t-PA activity (Rs = 0.946, p < 0.01, data not shown).

**Table 4:** t-PA/plasmin activity in serum of stroke patients.

	t-PA activity (RFU/min/μg prot)			Plasmin activity (RFU/min/μg prot)		
	Non-treated patients	rt-PA-treated patients	P value^a^	Non-treated patients	rt-PA-treated patients	P value^a^
**D0**	2.62 ± 0.25	3.35 ± 0.29	0.095	7.89 ± 1.29	10.71 ± 1.53	0.246
**D1**	2.77 ± 0.44	3.99 ± 0.22	0.063	9.50 ± 1.76	13.67 ± 1.49	0.098
**D7**	3.43 ± 0.55	4.52 ± 0.33	0.254	14.19 ± 3.03	15.84 ± 1.55	0.548
**D90**	2.84 ± 0.41	4.02 ± 0.54	0.396	10.32 ± 1.92	14.32 ± 2.62	0.700

Serum t-PA and plasmin activity in stroke patients (non-treated, n = 12 and rt-PA-treated, n = 23) assessed at D0 (admission), D1, D7 and D90 after ischemic stroke. Values are expressed as mean ± SEM.

^a^ Differences between the two groups of patients were analyzed at the different time points using the non-parametric Mann–Whitney-U test with significance set at (*) p <0.05.

Concerning potential association between serum BDNF levels and concurrent t-PA or plasmin activity in the whole cohort of patients and separately in rt-PA-treated or in non-treated patients (Table 5), our results showed a positive correlation between serum BDNF levels and t-PA or plasmin activity at day 1 (Rs = 0.582, p = 0.002 and Rs = 0.551, p = 0.006, respectively) in the whole cohort of patients (Rs = 0.582, p = 0.002 and Rs = 0.551, p = 0.006, respectively) and in the non-treated group of patients (Rs = 0.661, p = 0.033 and Rs = 0.685, p = 0.0025, respectively).

**Table 5:** Correlations between serum BDNF and t-PA/plasmin activity in stroke patients.

	All patients (n = 29)				Non-rt-PA-treated patients (n = 11)				rt-PA-treated patients (n = 18)			
	D0	D1	D7	D90	D0	D1	D7	D90	D0	D1	D7	D90
**BDNF vs t-PA activity**	Rs = 0.067 p = 0.736^a^	Rs = 0,582 **p = 0.002** ^b^*	Rs = -0.191p = 0.441^**b**^	Rs = 0,299p = 0.299^a^	Rs = 0.054p = 0.865^b^	Rs = 0.661 **p = 0.033** ^b^*	Rs = -0.107p = 0.781^b^	Rs = 0.829p = 0.053^b^	Rs = -0.088p = 0.727^a^	Rs = -0.033p = 0.890^a^	Rs = -0.189 p = 0.578 ^a^	Rs = 0.355 p = 0.388 ^a^
**BDNF vs plasmin activity**	Rs = 0.059 p = 0.766^b^	Rs = 0.163 **p = 0.015** ^a^	Rs = -0.254p = 0.309^a^	Rs = 0.152p = 0.604^a^	Rs = -0.091p = 0.785^b^	Rs = 0.685 p = **0.025** ^**b**^	Rs = -0.107p = 0.781^b^	Rs = 0.829p = 0.053^b^	Rs = 0.022p = 0.928^b^	Rs = 0.157p = 0.540^b^	Rs = -0.236 p = 0.467 ^b^	Rs = -0.452 p = 0.233 ^b^

BDNF levels and t-PA/plasmin activity were measured at D0 (admission), D1, D7 and D90.

^a^Correlation between the two parameters was evaluated by using the Pearson correlation test.

^b^Correlation between the two parameters was evaluated using the Spearman correlation test. Significance level (*) p <0.05.

#### Impact of rt-PA treatment in neurological recovery

The reduction in the NIHSS score from day 0 to day 1, to day 7 and to day 90 (Table 6) was significantly greater in rt-PA-treated than in non-treated patients. However, as shown in Table 7, serum BDNF at day 0 did not correlate with NIHSS score reduction occurring during the periods day 0–1, day 0–7 and day 0–90 periods when all of the patients were taken into consideration. Similarly, serum BDNF levels at day 1 did not correlate with NIHSS score reduction occurring during the periods day 0–7 and day 0–90 periods and serum BDNF levels at day 7 did not correlate with neurological improvement occurring during the period day 0–90 (Table 7).

**Table 6:** NIHSS score reduction between non-treated and rt-PA-treated patients over periods ranging from D0 to D1, D0 to D7 and D0 to D90.

	Non-treated patients	rt-PA-Treated patients	P value^a^
NIHSS variations between D0-D1	0.222 ± 4.680	-3.500 ± 2.747	0.032 *
NIHSS variations between D0-D7	-1.857 ± 2.116	-5.625 ± 2.669	0.01 *
NIHSS variations between D0-D90	-4.833 ± 3.189	-10.333 ± 3.204	0.041 *

NIHSS score reduction between non-treated (n = 14) and rt-PA-treated patients (n = 24) over periods ranging from D0 to D1, D0 to D7 and D0 to D90. Values are expressed as mean ± SEM. Differences between the two groups of patients were analyzed at the different time points using the non-parametric Mann–Whitney-U test with significance set at (*) p <0.05. NIHSS = National Institute of Health Stroke Scale.

**Table 7:** Correlations between NIHSS score variations over periods ranging from D0 to D1, D0 to D7 and D0 to D90 and serum BDNF levels in stroke patients at D0, D1, and D7.

	Serum BDNF levels		
	D0	D1	D90
NIHSS variations between D0-D1	Rs = -0.105 p = 0.705	-	-
NIHSS variations between D0-D7	Rs = -0.460 p = 0.181	Rs = -0.021 p = 0.939	-
NIHSS variations between D0-D90	Rs = -0.444 p = 0.159	Rs = -0.091 p = 0.776	Rs = -0.312 p = 0.381

Correlations between NIHSS score variations over periods ranging from D0 to D1, D0 to D7 and D0 to D90 and serum BDNF levels in stroke patients (non-treated + rt-PA-treated, n = 38) at D0, D1, and D7. Significance level p <0.05. NIHSS = National Institute of Health Stroke Scale.

#### Correlation between serum BDNF levels and cardiovascular score

As shown in Table 8, the cardiovascular score at hospital admission (a high score meaning a high risk) correlated negatively with serum BDNF levels at day 0 and day 90 (Rs = -0.450, p = 0.035; Rs = -0.508, p = 0.043; respectively) in the whole cohort of patients only. At the intermediate time points (day 1 and day 7) and in the separate groups of patients (non-treated and rt-PA-treated), no association could be found.

**Table 8:** Correlation between serum BDNF and cardiovascular score in stroke patients.

	All patients (n = 28)				non-treated patients (n = 10)				rt-PA-treated patients (n = 18)			
	D0	D1	D7	D90	D0	D1	D7	D90	D0	D1	D7	D90
**BDNF vs Cardio-vascular score**	Rs = -0.450 p = 0.035* ^b^	Rs = -0.097 p = 0.617^**b**^	Rs = -0.056 p = 0.818^**b**^	Rs = -0.508 **p = 0.043** ^**b**^ *	Rs = -0.559 p = 0.081 ^b^	Rs = -0.188 p = 0.583 ^b^	Rs = -0.288 p = 0.491 ^b^	Rs = -0.126 p = 0.720 ^b^	Rs = 0.089 p = 0.717 ^b^	Rs = 0.105 p = 0.674 ^b^	Rs = 0.442 p = 0.159 ^b^	Rs = -0.424 p = 0.178 ^b^

Correlations between serum BDNF levels and cardiovascular score in stroke patients. Serum BDNF levels were measured at D0 (admission), D1, D7 and D90 and correlated with cardiovascular score at admission. Significance level (*) p <0.05.

^b^Correlation between the two parameters was evaluated using the Spearman correlation test. Significance level (*) p <0.05.

### Preclinical study

#### Effect of rt-PA treatment on serum BDNF levels in stroke rats

**Fig 2:**
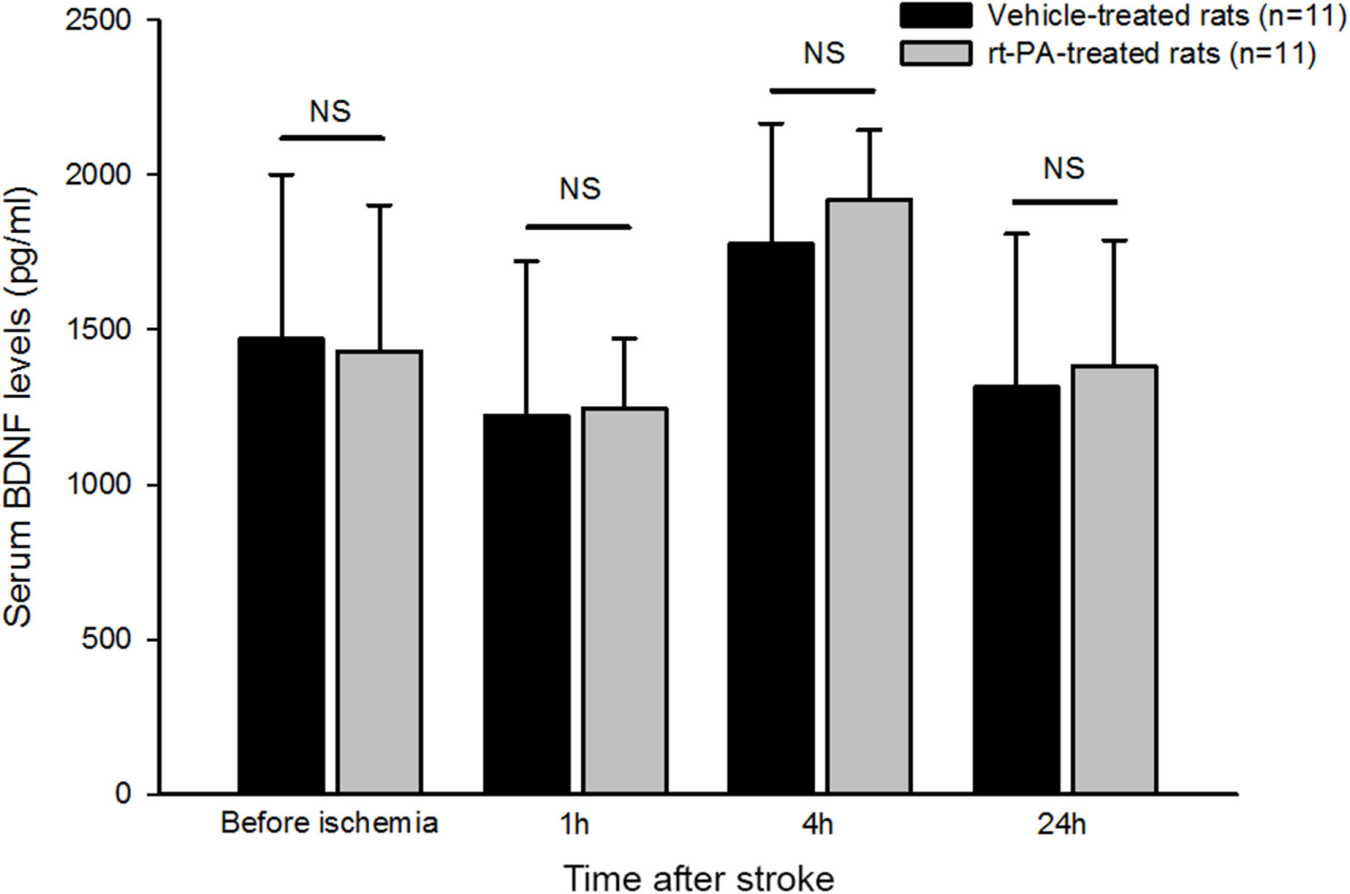
Comparison of serum BDNF levels in stroke rats treated or not with rt-PA. BDNF levels were assessed before and after (1h, 4h and 24h) photothrombotic stroke induction. Values are expressed as mean ± SEM. ^a^Differences between two groups of rats were analyzed at the different time points using the non-parametric Mann–Whitney-U test with significance set at (*) p <0.05.

Serum BDNF levels were measured before and after (1h, 4h and 24h) the induction of stroke in rats treated with rt-PA or vehicle. The results showed that that no statistical difference in serum BDNF was found between the two groups of animals whatever the time points considered (Fig 2).

#### Effect of rt-PA treatment on serum t-PA and plasmin activity in stroke rats and correlations between serum BDNF levels and t-PA/plasmin activity

Serum t-PA and plasmin activity were measured before and after (1h, 4h and 24h) the induction of stroke in rats treated with rt-PA or vehicle. In vehicle-treated animals, no difference in t-PA and plasmin activity was detected at the different time points (data not shown). By contrast, rt-PA-treated stroke rats exhibited higher t-PA and plasmin activity at 1h post-stroke (i.e. immediately at the end of rt-PA perfusion) as compared to vehicle-treated animals (+513%, p = 0.003; +6543%, p = 0.003, respectively. Data not shown). No association was detected between serum BDNF levels and t-PA activity or between serum BDNF levels and plasmin activity (Table 9) whatever the time point considered. As expected, in the whole cohort of animals, plasmin activity correlated significantly with t-PA activity (Rs = 0.900, p < 0.01, data not shown).

**Table 9:** Correlations between serum BDNF levels and t-PA/plasmin activity in stroke rats.

	All rats (n = 22)				vehicle-treated rats (n = 11)				rt-PA-treated rats (n = 11)			
	T0	T1	T4	T24	T0	T1	T4	T24	T0	T1	T4	T24
**BDNF vs t-PA activity**	Rs = 0.467 p = 0.186^b^	Rs = 0,550 p = 0.111^b^	Rs = -0.168 p = 0.665^a^	Rs = -0,510 p = 0.109^a^	Rs = -0.876 p = 0.124^a^	Rs = -0.894 p = 0.106^a^	Rs = -0.300 p = 0.700^a^	Rs = -0.378 p = 0.531^a^	Rs = 0.400 p = 0.517^b^	Rs = 0.500 p = 0.450^b^	Rs = -0.300 p = 0.683^b^	Rs = -0.029 p = 1.000^b^
**BDNF vs plasmin activity**	Rs = -0.008 p = 0.983^a^	Rs = 0.456 p = 0.186^b^	Rs = 0.530 p = 0.142^a^	Rs = -0.098 p = 0.787^a^	Rs = 0.059 p = 0.941^a^	Rs = 0.452 p = 0.548^a^	Rs = -0.429 p = 0.571^a^	Rs = 0.411 p = 0.589^a^	Rs = -0.100 p = 0.950^b^	Rs = 0.100 p = 0.950^b^	Rs = -0.200 p = 0.783^b^	Rs = -0.371 p = 0.497^b^

Correlations between serum BDNF levels and t-PA/plasmin activity in stroke rats. BDNF levels and t-PA/ plasmin activity were measured before and after (1h, 4h and 24h) photothrombotic stroke induction.

^a^Correlation between the two parameters was evaluated using the Pearson correlation test. Significance level (*) p <0.05.

^b^Correlation between the two parameters was evaluated using the Spearman correlation test. Significance level (*) p <0.05.

## Discussion

Our study revealed higher serum BDNF levels in the acute period following hospital admission (from day 1 to day 7) and better neurological recovery in rt-PA-treated than in non-treated stroke patients. However, serum BDNF levels at admission (day 0) or at days 1, 7 and 90 did not correlate with neurological improvement. Besides, negative correlations were identified between the cardiovascular score at day 0 and serum BDNF levels at days 0 and 90.

The recombinant form of t-PA is the gold standard treatment of cerebral artery thrombosis. t-PA perfusion exhibits an absolute benefit ranging from 11% to 13% depending on the stroke outcome scale used [7]. Thus, the small number of patients enrolled in our study may explain why the NIHSS score at a given time point did not differ between rt-PA-treated and non-treated stroke patients. Nevertheless, our results showed that the reduction in the NIHSS score occurring during the periods day 0–1, day 0–7 and day 0–90 periods was significantly greater in patients treated with rt-PA than in non-treated patients, thus supporting a beneficial effect of rt-PA treatment on stroke outcome, even in a small cohort of stroke patients.

Circulating BDNF levels in stroke patients are poorly documented. Plasma BDNF levels were reported to remain stable from hospital admission to day 7 [22]. However, circulating BDNF levels at both the acute (day 1) and chronic (3–6 months) stages of stroke were lower in patients with post-stroke depression (PSD) than in non-PSD patients [23,24]. From these data, serum BDNF levels were expected to mirror brain BDNF levels after stroke. Indeed, there is a consensus that both circulating and brain BDNF levels are decreased in depression [25]. The present study which is the first to be designed to assess the impact of rt-PA treatment on circulating BDNF levels in stroke patients, revealed higher serum BDNF levels in rt-PA treated than in non-treated stroke patients at least from day 1 to 7 after stroke onset. In light of this result, it would have been tempting to consider post-stroke serum BDNF as a biomarker of stroke recovery. However, given the absence of a correlation between neurological outcomes and serum BDNF levels, measuring BDNF levels in serum may not be useful to predict the recovery in stroke patients. The unresolved point concerns the mechanisms involved in the higher serum BDNF levels in rt-PA-treated patients as compared to non-treated patients. Although speculative, two different mechanisms can be proposed. The higher serum BDNF levels in rt-PA-treated patients than in non-treated patients could reflect a plasmin-dependent transformation of circulating pro-BDNF to mature BDNF. Consistently, pro-BDNF has recently been detected in the blood [26]. However, in contradiction to this mechanism, there was no correlation between serum BDNF levels and t-PA/plasmin activity in either stroke patients (except at day 1 for the whole cohort of patients) or in stroke rats. In addition, in the analysis by groups, no association was found between these parameters in rt-PA-treated patients or animals. Moreover, given the doubtful reliability of ELISA kits to discriminate between pro-BDNF, the BDNF prodomain and the mature form of BDNF, it would be difficult to confirm that such a mechanism was involved. A second hypothesis is that higher serum BDNF levels in rt-PA-treated than in non-treated patients could reflect elevated brain BDNF levels and the subsequent secretion of BDNF into the blood in treated patients. Consistently, rt-PA administration induces an increase in brain BDNF levels [13] and BDNF can cross the blood brain barrier from the brain to the blood [27] especially since rt-PA has been shown to exacerbate stroke-induced blood brain barrier disruption [7]. However, the fact that serum BDNF levels did not correlate with concurrent brain BDNF levels in rats subjected to hemispheric embolization with microspheres [17] raises questions about the contribution of brain-derived BDNF to the elevation of serum BDNF levels observed in rt-PA-treated patients. Besides, it has to be kept in mind that serum BDNF levels are influenced by numerous factors including gender, platelet counts, Val66Met polymorphism, smoking status, depression and age [23,24,28–33], and that these factors may be confounding factors when post-stroke serum BDNF is used as an index of brain BDNF levels.

Cardiologists are showing increasing interest in circulating BDNF as a potential biomarker of cardiovascular health [34–38]. In addition, recent data highlight the use of circulating BDNF as a predictor of cardiovascular events. For example, plasma BDNF was found to be an independent predictor of 4-year major coronary events in patients with angina pectoris [39] and low serum BDNF levels have recently been reported to be predictive of vascular brain injury [40]. In addition, preclinical evidence suggests that circulating BDNF levels could be considered an index of endothelial BDNF expression. First, endothelial cells from peripheral or central vessels have been shown to express BDNF [21,41,42]. Secondly, physical training, which has been associated with high serum BDNF levels in humans [43], results in BDNF overexpression by the endothelium of both peripheral [44] and cerebral vessels [21] in rodents, while hypertension [44] and type 2 diabetes [45] are associated with decreased BDNF expression in peripheral and cerebral endothelium, respectively. Overall, the data led us to suspect a link between cardiovascular status and serum BDNF levels in stroke patients and in turn to investigate the relationship between the European cardiovascular risk score at admission (a high score meaning a high risk) and serum BDNF levels. Our results in stroke patients showing a negative correlation between this score and BDNF levels at admission and at day 90 and our data in stroke rats showing a lack of any difference in serum BDNF levels between rt-PA- and vehicle-treated animals come in support of a connection between post-stroke BDNF levels and cardiovascular status. Indeed, unlike stroke patients, stroke rats had no cardiovascular risk factors. Thus, circulating BDNF levels could also be related to vascular BDNF expression and mirror the cardiovascular status. This negative correlation is indeed in line with a recent study showing that serum BDNF levels were inversely associated with cardiovascular disease and mortality [46] and with risk of incident stroke or transient ischemia attack [40].

In conclusion, despite evidence of higher circulating BDNF levels and better outcome in rt-PA-treated than in non-treated stroke patients, our results do not support the use of serum BDNF as a biomarker of stroke outcome. They rather highlight cardiovascular risk as a potential confounding factor when circulating BDNF is used to investigate levels of BDNF in the brain.

## Supporting Information

S1 Clinical Data (XLSX)

S1 Table Correlation between serum BDNF and blood pressure (BP) at admission. Serum BDNF levels were assessed at D0 (admission), D1, D7 and D90 and correlated with blood pressure (BP) measured at admission. ^a^Correlation between the two parameters was evaluated by using the Pearson correlation test. Significance level (*) p <0.05. ^b^Correlation between the two parameters was evaluated by using the Spearman correlation test. Significance level (*) p <0.05.
(DOCX)

S2 Table Impact of time-to-treatment interval on serum BDNF levels at D0 and D1. Serum BDNF levels at D0 (admission) and D1 in patients treated with rt-PA before and beyond 2h after the stroke onset. Values are expressed as means ± SEM. ^a^ Differences between the two groups of patients at the different time points were analyzed using the non-parametric Mann–Whitney-U test with the significance set at (*) p <0.05.
(DOCX)
