# Supplementary material for: Relevance of Post-Stroke Circulating BDNF Levels as a Prognostic Biomarker of Stroke Outcome. Impact of rt-PA Treatment
Source: PLoS One. 2015 Oct 15;10(10):e0140668. doi: 10.1371/journal.pone.0140668 (PMC4607484; doi:10.1371/journal.pone.0140668)
Supplement: S1 Table — Serum BDNF levels were assessed at D0 (admission), D1, D7 and D90 and correlated with blood pressure (BP) measured at admission. aCorrelation between the two parameters was evaluated by using the Pearson correlation test. Significance level (*) p <0.05. bCorrelation between the two parameters was evaluated by using the Spearman correlation test. Significance level (*) p <0.05. (DOCX) [file pone.0140668.s002.docx]

**S1 Table. Correlation between serum BDNF and blood pressure (BP) at admission.**

|  | All patients (n=28) | | | | non-treated patients (n=10) | | | | rt-PA-treated patients (n=18) | | | |
| --- | --- | --- | --- | --- | --- | --- | --- | --- | --- | --- | --- | --- |
|  | **D0** | **D1** | **D7** | **D90** | **D0** | **D1** | **D7** | **D90** | **D0** | **D1** | **D7** | **D90** |
| SerumBDNF vs BP | Rs = -0.023 p = 0.906^b^ | Rs = -0.020 p = 0.917^b^ | Rs = -0.010 p = 0.961^b^ | Rs = -0.190 p = 0.441^b^ | Rs = -0.373 p = 0.289^a^ | Rs = -0.366 p = 0.298^a^ | Rs = -0.418 p = 0.350^a^ | Rs = -0.095 p = 0.840^a^ | Rs = 0.284 p = 0.248^b^ | Rs = 0.054 p = 0.824^b^ | Rs = 0.548 p = 0.076^b^ | Rs = -0.370 p = 0.245^b^ |

Serum BDNF levels were assessed at D0 (admission), D1, D7 and D90 and correlated with blood pressure (BP) measured at admission.

^a^Correlation between the two parameters was evaluated by using the Pearson correlation test. Significance level (*) p <0.05.

^b^Correlation between the two parameters was evaluated by using the Spearman correlation test. Significance level (*) p <0.05.
