# Supplementary material for: Relevance of Post-Stroke Circulating BDNF Levels as a Prognostic Biomarker of Stroke Outcome. Impact of rt-PA Treatment
Source: PLoS One. 2015 Oct 15;10(10):e0140668. doi: 10.1371/journal.pone.0140668 (PMC4607484; doi:10.1371/journal.pone.0140668)
Supplement: S2 Table — Serum BDNF levels at D0 (admission) and D1 in patients treated with rt-PA before and beyond 2h after the stroke onset. Values are expressed as means ± SEM. a Differences between the two groups of patients at the different time points were analyzed using the non-parametric Mann–Whitney-U test with the significance set at (*) p <0.05. (DOCX) [file pone.0140668.s003.docx]

**S2 Table. Impact of time-to-treatment interval on serum BDNF levels at D0 and D1.**

|  | Serum BDNF levels (pg/ml) | | |
| --- | --- | --- | --- |
|  | Time-to-treatment interval <120min (n = 7) | Time-to-treatment interval >120min (n = 17) | P value^a^ |
| D0 | 12291.43 ± 613.64 | 9738.71 ± 935.05 | 0.556 |
| D1 | 11195.36 ± 1366.60 | 10495.18 ± 996.61 | 0.441 |

Serum BDNF levels at D0 (admission) and D1 in patients treated with rt-PA before and beyond 2h after the stroke onset. Values are expressed as means ± SEM.

^a^ Differences between the two groups of patients at the different time points were analyzed using the non-parametric Mann–Whitney-U test with the significance set at (*) p <0.05.
